# Predicting tracheostomy in multiple injured patients with severe thoracic injury (AIS ≥ 3) with the new T_3_P-Score: a multivariable regression prediction analysis

**DOI:** 10.1038/s41598-023-30461-x

**Published:** 2023-02-24

**Authors:** Felix M. Bläsius, Sebastian Wutzler, Philipp Störmann, Thomas Lustenberger, Michael Frink, Marc Maegele, Matthias Weuster, Jörg Bayer, Klemens Horst, Michael Caspers, Andreas Seekamp, Ingo Marzi, Frank Hildebrand, Hagen Andruszkow

**Affiliations:** 1 Department of Orthopaedics, Trauma and Reconstructive Surgery, University Hospital RWTH Aachen, Pauwelsstraße 30, 52074 Aachen, Germany; 2 Department of Trauma, Hand, and Orthopaedic Surgery, Helios Dr. Horst Schmidt Kliniken Wiesbaden, Ludwig-Erhard-Straße 100, 65199 Wiesbaden, Germany; 3 Department of Trauma, Hand, and Reconstructive Surgery, University Hospital Frankfurt/Main, Theodor-Stern Kai 7, 60590 Frankfurt am Main, Germany; 4 Center for Orthopaedics and Trauma Surgery, University Hospital Giessen and Marburg GmbH, Marburg, Baldingerstraße, 35043 Marburg, Germany; 5 Department of Traumatology and Orthopaedic Surgery Cologne-Merheim Medical Centre (CMMC), Institute for Research in Operative Medicine (IFOM), Experimental/Clinical Research Unit, University Witten-Herdecke, 51109 Cologne, Germany; 6 Clinic for Trauma Surgery, Diako Hospital Flensburg, 24939 Flensburg, Germany; 7 Department of Orthopaedics and Trauma Surgery, Medical Centre Albert-Ludwigs-University of Freiburg, Sir-Hans-A.-Krebs-Straße, 79106 Freiburg, Germany; 8 Department of Trauma Surgery, University Hospital Schleswig-Holstein, Campus Kiel, 24105 Kiel, Germany

**Keywords:** Medical research, Outcomes research

## Abstract

Multiple trauma patients with severe chest trauma are at increased risk for tracheostomy. While the risk factors associated with the need for tracheostomy are well established in the general critical care population, they have not yet been validated in a cohort of patients suffering severe thoracic trauma. This retrospective cohort study analysed data on patients aged 18 years or older who were admitted to one of the six participating academic level I trauma centres with multiple injuries, including severe thoracic trauma (AIS_Thorax_ ≥ 3) between 2010 and 2014. A multivariable binary regression was used to identify predictor variables for tracheostomy and to develop the Tracheostomy in Thoracic Trauma Prediction Score (T_3_P-Score). The study included 1019 adult thoracic trauma patients, of whom 165 underwent tracheostomy during their intensive care unit (ICU) stay. Prehospital endotracheal intubation (adjusted OR [AOR]: 2.494, 95% CI [1.412; 4.405]), diagnosis of pneumonia during the ICU stay (AOR: 4.374, 95% CI [2.503; 7.642]), duration of mechanical ventilation (AOR: 1.008/hours of intubation, 95% CI [1.006; 1.009]), and an AIS_Head_ ≥ 3 (AOR 1.840, 95% CI [1.039; 3.261]) were independent risk factors for tracheostomy. Patients with sepsis had a lower risk of tracheostomy than patients without sepsis (AOR 0.486, 95% CI [0.253; 0.935]). The T_3_P-Score had high predictive validity for tracheostomy (ROC_AUC_ = 0.938, 95% CI [0.920, 0.956]; Nagelkerke’s R^2^ was 0.601). The T_3_P-Score’s specificity was 0.68, and the sensitivity was 0.96. The severity of thoracic trauma did not predict the need for tracheostomy. Follow-up studies should validate the T_3_P-Score in external data sets and study the reasons for the reluctant use of tracheostomy in patients with severe thoracic trauma and subsequent sepsis.

**Trial registration: **The study was applied for and registered a priori with the respective ethics committees.

## Introduction

Multiple trauma patients with relevant thoracic trauma are at increased risk for prolonged mechanical ventilation compared to patients without severe thoracic injuries^1^. Therefore, it is of particular importance to determine which independent influencing factors in this vulnerable patient group increase the risk of prolonged ventilation and the likelihood of requiring tracheostomy. Tracheostomies are routinely performed in intensive care unit (ICU) patients requiring prolonged mechanical ventilation to prevent endotracheal tube-associated complications, such as ulceration, vocal cord paralysis, and laryngotracheal stenosis^2,3^. At the same time, tracheostomy is considered a relatively safe procedure, with a residual risk of severe complications, such as tension pneumothorax or injury to the aortic arch. The extent to which a tracheostomy can positively influence the course of a critically ill patient has been the subject of controversial discussions^2^. Tracheostomy improves oral hygiene, patient communication, facilitates weaning, and improves patient comfort when compared to endotracheal intubation^3^.

To date, few RCTs have contributed to evidence-based recommendations on which patients should receive tracheostomy and when. No RCTs on vulnerable subgroups (e.g. thoracic trauma patients) are available^2^. Due to the low number of studies, the recommendations of expert committees are used to decide on tracheostomies^4^. In the German S2k guideline of “prolonged weaning” by the German Respiratory Society, it is recommended to discuss the performance of a tracheostomy in patients after “clinical estimation of prolonged weaning” with concomitant inability of non-invasive ventilation 4–7 days after intubation. In this context, clinical estimation of prolonged weaning is defined as more than three spontaneous breathing attempts (SBA) or > 7 days after the last SBA^5^. Moreover, the Brazilian recommendations of mechanical ventilation 2013 highlight the early clinical estimation of prolonged weaning in three specific risk groups (severe polytrauma, high spinal cord injury, and severe traumatic brain injury [TBI]) without further specifications on when and how a tracheostomy should be performed^6^. The estimation of a prolonged weaning is still difficult, even for experts, as shown by high-quality RCTs, which challenges the current practice of "clinical estimation of prolonged weaning"^7^. Overall, there are currently primarily expert opinions and there is an urgent need for more evidence in this field. For this reason, we studied a typical risk group for prolonged mechanical ventilation: severely injured patients with severe thoracic trauma. The results were used to develop a tracheostomy prediction score for thoracic trauma patients, which can be used for the early identification of patients who have a high probability that a tracheostomy will be necessary in the further ICU stay to support clinical decision making and for research purposes. Therefore, we performed a retrospective data analysis using a comprehensive thoracic trauma database. A multivariable logistic regression analysis was performed to achieve the above-described objective.

## Patients and methods

This study was conducted by the Trauma Section of the German Interdisciplinary Association for Intensive and Emergency Medicine (Deutsche Interdisziplinäre Vereinigung für Intensiv- und Notfallmedizin, DIVI). In December 2015, the section embarked on a retrospective, observational study of the quality of care in patients with thoracic trauma (AIS_Thorax_ ≥ 3) who underwent mechanical ventilation (MV) from 2010 to 2014. This study was part of a larger research project with the aim of improving the treatment of multiple injured patients with thoracic trauma, which was previously described by Wutzler et al.^8^. Six German university hospitals (Aachen, Cologne, Frankfurt, Freiburg, Kiel, and Marburg) contributed patient data for analysis. All participating hospitals were academic level I trauma centres.

This study follows the guidelines of the revised UN Declaration of Helsinki in 1975 and its latest amendment in 2013 (64th General Assembly). The following approvals were provided by each institution’s ethical committee: Independent Ethics Committee of the University RWTH Aachen: EK 346/15, Ethics Commission of the University of Cologne 18/2016, Ethics Committee Office of the University of Frankfurt: 220/16, Ethics Committee of the University of Kiel: B 248/16, Ethics Committee of the University of Freiburg: 275/16, Ethics Committee of the University of Marburg: No ethics committee vote necessary for the retrospective analysis. Due to the study’s retrospective nature, informed consent from the study participants was waived in accordance with the ethical approval from Independent Ethics Committee of the University RWTH Aachen. The other above mentioned ethics committees and commissions confirmed this decision.

The reporting was in accordance with the recommendations of the TRIPOD statement (v2015)^9^.

### Implementation of the clinical database

Details of the database^10^ used are given in Supplement 1.

#### Definitions and diagnosis criteria

Overall injury severity was calculated by the Injury Severity Score (ISS), as described by Baker et al.^11^. The Abbreviated Injury Scale (AIS, Version 2005/Update 2008, Association for the Advancement of Automotive Medicine, Barrington, IL) was used as a global system for injury coding and severity classification. The severity of injuries was recorded according to the AIS as 1 (minor), 2 (moderate), 3 (severe, not life-threatening), 4 (serious, life-threatening), 5 (critical, survival uncertain), and 6 (maximum, currently untreatable). The decision to perform a tracheostomy was made by interdisciplinary teams (intensivists, trauma surgeons, and neurosurgeons). Multiple organ failure was diagnosed at any time during the hospitalization according to the Sequential Organ Failure Assessment (SOFA), where 3 or 4 points for an organ was considered as organ failure^12^. Sepsis was based on the sepsis-3 definition^13^. The patients’ physical status was graded using the ASA classification system on admission^14^.

The Clinical Pulmonary Infection Score (CPIS) was used to assess the risk of pneumonia in ventilated patients^15^. A total of > 6 points was accepted as pneumonia. In non-ventilated patients, pneumonia was defined as the presence of a new progressive infiltrate accompanied by at least two of the following symptoms:

- Purulent respiratory secretions
- Body temperature ≥ 38 °C or ≤ 35 °C
- Leucocytosis (white blood cell count of ≥ 10,000/mm^3^) or leucopoenia (white blood cell count of ≤ 4500/mm^3^, or more than 15% immature neutrophils)

### Statistics

Continuous values are presented as mean, standard deviation (SD), and range [X, Y], where applicable. Differences in categorical and continuous variables were evaluated by a chi-square test and a Mann–Whitney U test, respectively. The significance level was set at α = 0.05 (two-sided *p*-value). All statistical analyses were performed using the Statistical Package for Social Sciences (SPSS 28.0; IBM Inc., Armonk, NY, USA). Calibration curves were calculated using SAS 9.4 (TS Level 1M4, SAS Institute, Cary, NC, USA).

#### Model A: Multivariable binary logistic regression analysis

The binary logistic regression model was fit using the dichotomous variable “tracheostomy” as the dependent endpoint. Univariable logistic regression analyses of multiple variables were conducted to identify independent variables for inclusion in the multivariable logistic regression model (Table 2). The variables included age (years), sex (m/f), ASA classification (1/2/3/4/5), AIS_Thorax_ categories, AIS_Head_ ≥ 3 (y/n)_,_ ISS categories (9–15/16–31/32–75), prehospital endotracheal intubation (y/n), duration of mechanical ventilation (hours), aspiration (y/n), pneumonia (y/n), multiple organ failure (y/n), and sepsis (y/n). The significance level for considering variables from the univariable analyses for the multivariable analysis was set at *α* = 0.05. The statistical significance of each regression coefficient was tested using the Wald test. The significance level for the interpretation in the multivariable regression analysis was set at *α* = 0.05. The goodness of fit was measured using ROC_AUC_, the Hosmer–Lemeshow test (*X*^2^), and the omnibus test. A calibration curve was produced which contrasts tracheostomy probabilities observed in the data with those estimated from the T_3_P-Score logistic regression model. Observed probabilities were smoothed by LOESS (*k* = 0.75) as recommended by Austin et al.^16^. The overall performance of the model was evaluated using Nagelkerke’s R^2^. The internal validation of the results was performed using bootstrapping (1000 replications, bias-corrected, and accelerated [BCa]), as recommended by Steyerberg et al.^17^. The collinearity analysis was performed by evaluating the tolerance and the variance inflation factor (VIF). Odds ratios (OR) and adjusted odds ratios (AOR) were reported with the associated 95% confidence interval (CI).

#### Model B: Tracheostomy in Thoracic Trauma Prediction Score (T_3_P-Score)

We repeated the multivariable logistic regression with the aim of developing the T_3_P-Score. The variable "duration of MV" was transformed into “duration of MV groups” and quantified using categorical regression (0–67/68–180/181–299/300–440/441–1258) for higher robustness. The variables AIS_Head_ ≥ 3 (y/n), duration of MV groups (0–67/68–180/181–299/300–440/441–1258 in hours), prehospital endotracheal intubation (y/n), pneumonia (y/n), and sepsis (y/n) were included in the score due to their performance in the model A (*p* < 0.05). Quality assurance of the score (goodness of fit, collinearity analysis, performance, and validation) was performed as previously described. The predicted probability can be calculated according to the general principles of logistic regression^18^.

### Ethics approval and consent to participate

The following approvals were provided by each institution's ethical committee: Independent Ethics Committee of the University RWTH Aachen: EK 346/15, Ethics Commission of the University of Cologne 18/2016, Ethics Committee Office of the University of Frankfurt: 220/16, Ethics Committee of the University of Kiel: B 248/16, Ethics Committee of the University of Freiburg: 275/16, Ethics Committee of the University of Marburg: No ethics committee vote necessary for the retrospective analysis.

### Informed consent

All the study protocol was performed in accordance with the relevant guidelines and regulations.

## Results

**Figure 1:**
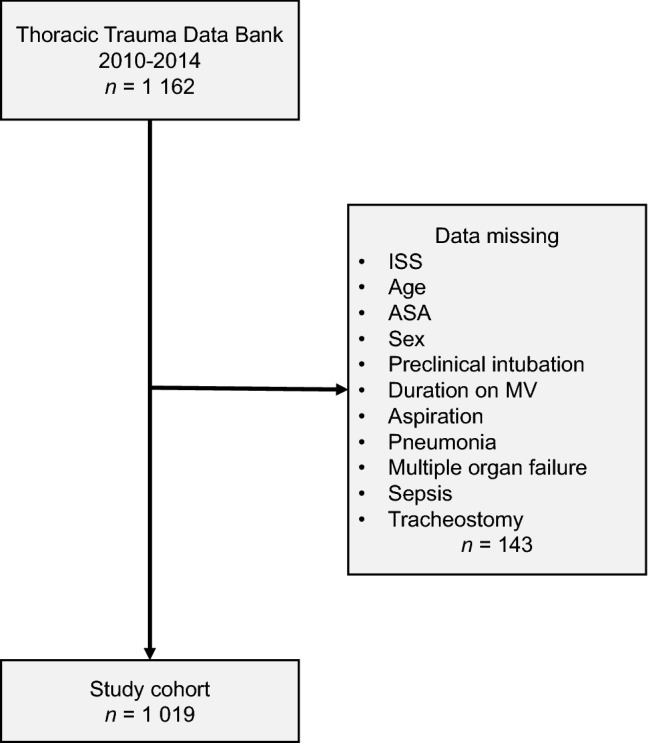
Flow chart indicating the inclusion and exclusion process.

A total of 1019 thoracic trauma patients were considered for comparison (Fig. 1). The mean age was 48.4 years (SD: 18.8 [18, 94]), and 76.0% were male. The cohort included 165 (16.2%) who underwent tracheostomies, and 854 (83.8%) who did not undergo tracheostomies. The detailed characteristics of the study patients are presented in Table 1.

**Table 1:** Characteristics.

	Total	Tracheostomy		*p*-value
	*n* = 1019	No *n* = 854	Yes *n* = 165	
Age	48.4 ± 18.8	48.0 ± 18.8	50.6 ± 18.8	< 0.001
Male (%)	76.0	75.1	80.6	0.127
ASA classification (%)				
1	58.3	59.1	54.2	0.336
2	32.8	32.3	35.3	
3	7.6	7.6	7.8	
4	1.3	1.0	2.6	
Blunt trauma (%)	96.7	96.2	99.4	0.038
ISS (pts.)	27.8 ± 12.6	26.2 ± 12.0	36.0 ± 12.5	< 0.001
AIS_Head_ ≥ 3 (%)	34.2	28.4	62.4	< 0.001
AIS_Thorax_ (%)				
3	58.0	60.5	44.8	< 0.001
4	25.7	25.4	27.3	
5	16.2	13.9	27.9	
6 (*n*)	1	1		
AIS_Abdomen_ ≥ 3 (%)	16.1	16.3	15.2	0.857
Prehospital endotracheal intubation (%)	44.5	37.8	78.8	< 0.001
Preclinical CPR (%)	3.7	3.1	7.5	0.009
Duration MV (h)	127.5 ± 203.3	67.7 ± 117.9	437.1 ± 263.7	< 0.001
Time-to-tracheostomy (days)			9.9 ± 7.8	
LOS (days)	20.3 ± 17.7	18.1 ± 16.0	32.1 ± 21.3	< 0.001
RISC II	12.2	10.4	21.5	< 0.001
Deceased (%)	8.8	9.3	6.7	0.284
NO (*n*)	6	1	5	
ECMO (*n*)	2	0	2	
Aspiration (%)	8.7	6.7	19.4	< 0.001
Pneumonia (%)	26.5	17.2	74.5	< 0.001
MOF (%)	14.3	10.5	33.9	< 0.001
Sepsis (%)	13.3	9.0	35.8	< 0.001

Trauma patients who underwent tracheostomies were older, had a higher mean ISS, and prehospital endotracheal intubation was more frequent compared to patients who did not undergo tracheostomies (Table 1). The mean time to tracheostomy (TTT) was 9.9 days. The relationship between the variables and tracheostomy is shown in Table 2. Patients’ characteristics that were associated with the performance of a tracheostomy were AIS_Thorax_ categories, AIS_Head_ ≥ 3, ISS groups, prehospital endotracheal intubation, duration of mechanical ventilation, aspiration, pneumonia, MOF, and sepsis (Table 2). Pneumonia represented the strongest risk factor in the univariable analysis.

**Table 2:** Univariable binary logistic regression analyses with “tracheostomy” as the dependent variable.

Variable	*p*-value	OR	95% CI
AIS_Head_ ≥ 3	< 0.001	4.149	2.967; 5.803
AIS_Abdomen_			
0	0.879		
1	0.999	0	0
2	0.528	0.873	0.572; 1.332
3	00.428	0.780	0.422; 1.442
4	0.492	1.286	0.628; 2.635
5	0.624	0.624	0.217; 2.501
AIS_Thorax_			
3 (reference)	< 0.001		
4	0.029	1.542	1.045; 2.274
5	< 0.001	2.701	1.804; 4.044
6	1.000	0	0
ISS			
9–15 (reference)	< 0.001		
16–31	0.052	2.122	0.993; 4.534
32–75	< 0.001	8.318	3.953; 17.502
Age	0.181	1.006	0.997; 1.015
ASA			
1 (reference)	0.322		
2	0.280	1.222	0.849; 1.758
3	0.590	1.191	0.631; 2.247
4	0.106	2.686	0.810; 8.908
Sex	0.152	0.745	0.499; 1.114
Prehospital endotracheal intubation	< 0.001	6.448	4.353; 9.552
Duration of MV	< 0.001	1.009	1.008; 1.011
Aspiration	< 0.001	3.862	2.441; 6.112
Pneumonia	< 0.001	14.699	10.035; 21.531
Multiple organ failure	< 0.001	4.489	3.089; 6.524
Sepsis	< 0.001	6.245	4.272; 9.130

### Model A: Multivariable logistic regression analysis

The AIS_Thorax_ categories, AIS_Head_ ≥ 3, ISS groups, prehospital endotracheal intubation, duration of MV, aspiration, pneumonia, multiple organ failure, and sepsis were identified by univariable regression for consideration in our multivariable analysis. Table 3 shows the AOR for these risk factors.

**Table 3:** Multivariable binary logistic regression model with “tracheostomy” as the dependent variable (model A).

Independent variables	Regression coefficient *β* *(bootstrap)*	BCa 95% CI for *β*	*Wald*	*p*-value	OR	95% CI	Collinearity statistics	
							Tolerance	VIF
AIS_Head_ ≥ 3	0.610	0.420; 1.277	4.366	0.037	1.840	1.039; 3.261	0.689	1.452
AIS_Thorax_ (pts.)							0.738	1.355
3 (reference)			0.415	0.937				
4	0.168	− 0.510; 0.783	0.259	0.611	1.182	0.620; 2.254		
5	0.203	− 0.583; 0.990	0.347	0.556	1.225	0.624; 2.404		
6	− 17.969	− 18.861; − 16.724	< 0.001	1.000	< 0.001	0		
ISS (pts.)							0.542	1.847
9–15 (reference)			0.938	0.626				
16–31	− 0.322	− 1.595; 1.501	0.305	0.581	0.725	0.231; 2.274		
32–75	− 0.063	− 1.472; 1.894	0.009	0.922	0.939	0.266; 3.321		
Prehospital endotracheal intubation	0.914	0.298; 1.523	9.922	0.002	2.494	1.412; 4.405	0.758	1.319
Duration of MV (h)	0.008	0.006; 0.011	99.352	< 0.001	1.008	1.006; 1.009	0.546	1.831
Aspiration	− 0.344	− 1.256; 0.375	0.846	0.358	0.709	0.341; 1.475	0.859	1.164
Pneumonia	1.476	0.856; 2.216	26.864	< 0.001	4.374	2.503; 7.642	0.599	1.668
Multiple organ failure	− 0.201	− 1.041; 0.546	0.434	0.510	0.818	0.450; 1.487	0.755	1.325
Sepsis	− 0.721	− 1.517; − 0.109	4.677	0.031	0.486	0.253; 0.935	0.648	1.543
Intercept	− 4.431	− 5.285; − 3.997	68.829	< 0.001	0.12			

Nagelkerke’s R^2^ was 0.602, ROC_AUC_ was 0.941, 95% CI [0.925, 0.958] (*p* < 0.001), the Hosmer–Lemeshow-Test was statistically significant (χ^*2*^ = 23.267, df = 8, *p* = 0.003), the omnibus test was statistically significant χ^2^ = 444.700, df = 12, *p* < 0.001.

**Figure 2:**
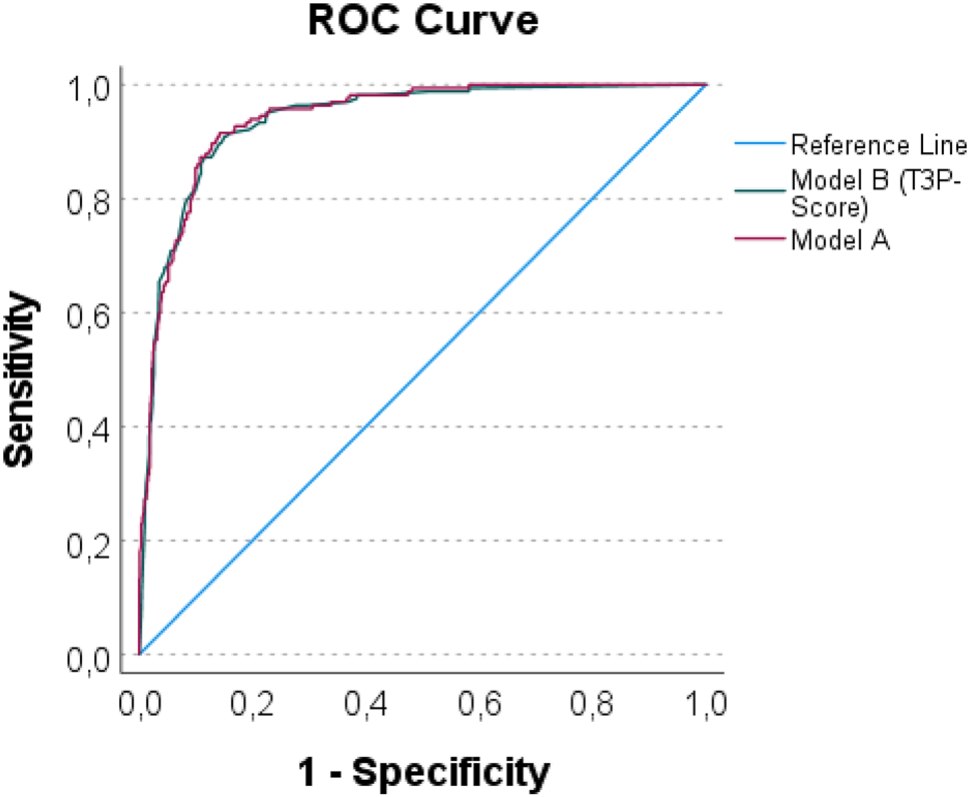
ROC_AUC_ curve of model A and model B (T_3_P-Score).

AIS_Head_ ≥ 3, prehospital endotracheal intubation, duration of, and pneumonia were independent variables increasing the risk of tracheostomy (Table 3). The presence of sepsis led to less frequent performance of tracheostomy. The predictive validity of the model was high (ROC_AUC_ = 0.941, 95% CI [0.925, 0.958], Table 3 and Fig. 2). The overall performance of Model A was 0.602 (Nagelkerke’s R^2^).

### Model B: The T_3_P-Score

**Figure 3:**
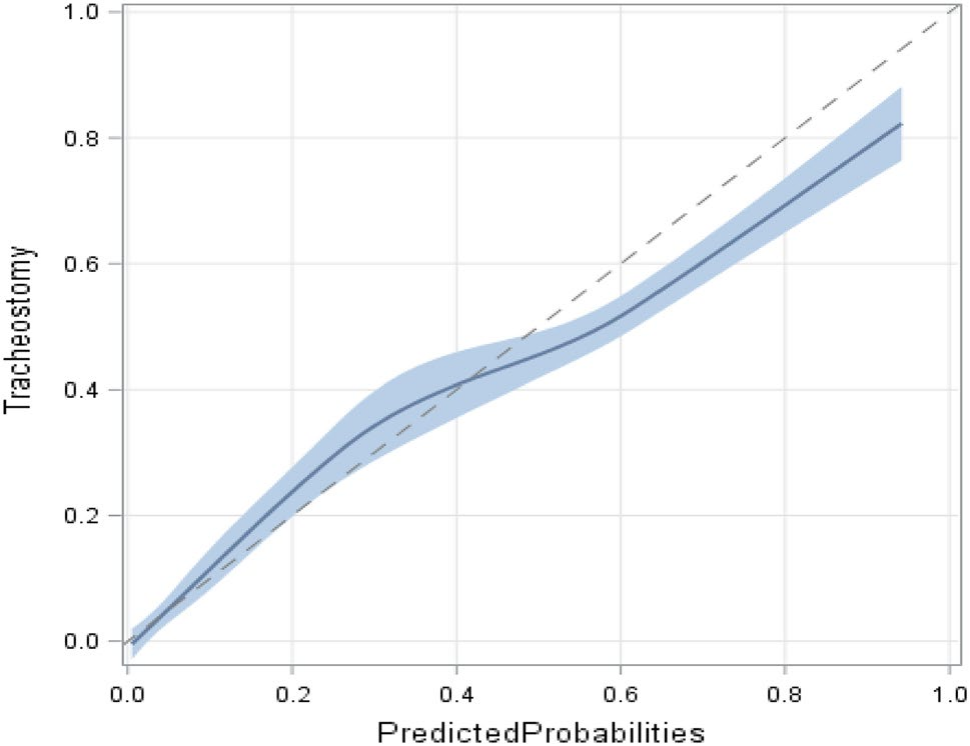
Calibration curve: LOESS smoothed observed probabilities vs. predicted probabilities for tracheostomy. Diagonal indicates ideal calibration.

The final T_3_P-Score model included AIS_Head_ ≥ 3, prehospital endotracheal intubation, duration of MV groups, pneumonia, and sepsis (Table 4). The T_3_P-Score predicts the need for tracheostomy (ROC_AUC_ = 0.938, 95% CI [0.920, 0.956]; Table 4 and Fig. 2). The overall performance of the model was 0.601 (Nagelkerke’s R^2^). The specificity and sensitivity were 0.96 and 0.68 (cut-off value: − 2.19), respectively. The calibration curve is displayed in Fig. 3.

**Table 4:** The T_3_P-Score (model B) for prediction of tracheostomy in thoracic trauma patients.

Independent variables	Regression coefficient *β (bootstrap)*	BCa 95% CI for *β*	*Wald*	*p*-value	OR	95% CI	Collinearity statistics	
							Tolerance	VIF
AIS_Head_ ≥ 3	0.645	0.108; 1.209	6.474	0.011	1.906	1.160; 3.132	0.848	1.179
Prehospital endotracheal intubation	0.813	0.238; 1.526	8.362	0.004	2.254	1.299; 3.911	0.789	1.267
Duration of MV (h)							0.531	1.884
0–67 (reference)			127.584	< 0.001				
68–180	0.892	0.046; 1.791	4.222	0.040	2.440	1.042; 5.714		
181–299	2.387	1.491; 3.555	34.154	< 0.001	10.882	4.887; 24.232		
300–440	3.312	2.313; 4.824	53.956	< 0.001	27.446	11.341; 66.421		
441–1258	4.718	3.632; 6.523	102.812	< 0.001	111.996	44.988; 278.811		
Pneumonia	1.259	0.520; 2.075	20.809	< 0.001	3.523	2.051; 6.051	0.607	1.649
Sepsis	− 0.674	− 1.359; − 0.061	4.927	0.026	0.510	0.281; 0.924	0.732	1.367
Intercept	− 4.650	− 5.291; -4.298	172.373	< 0.001	0.100			

Nagelkerke’s R^2^ was 0.601, ROC_AUC_ was 0.938, 95% CI [0.920, 0.956] (*p* < 0.001), the Hosmer–Lemeshow-test was not statistically significant (χ^*2*^ = 6.998, df = 7, *p* = 0.429), the omnibus test was statistically significant (χ^2^ = 443.948, df = 8, *p* < 0.001).

## Discussion

By combining the variables AIS_Thorax_ categories, AIS_Head_ ≥ 3, ISS groups, prehospital endotracheal intubation, duration of MV, aspiration, pneumonia, multiple organ failure, and sepsis, we were able to achieve a high predictive value for Model A. Moreover, after recalibration and focusing on a reduced variable set, the developed T_3_P Score showed a high specificity and sensitivity to predict a tracheostomy during the ICU stay of severely injured patients with severe thoracic trauma. The inclusion of 1069 patients provided a reliable basis of data, and the goodness of fit procedures demonstrated the high performance of the score.

As expected, severe TBI was a strong adjusted risk factor influencing the need for tracheostomy. This is in accordance with the current literature, according to which an early tracheostomy is associated with a better outcome (especially secondary complications) in TBI patients^19^. Therefore, current recommendations favour early tracheostomy in these patients, although this recommendation has been increasingly challenged, as the result of several randomised controlled studies with contradictory results^20^. In this context, the TracMan multicentre randomised controlled study reported no beneficial effect of an early tracheostomy vs. late tracheostomy on mortality, duration of ICU stay, or the hospital length of stay in 909 ICU patients^7^. In contrast, another multicentre randomised controlled study by Terragni et al. could observe a reduction in the duration of MV and the ICU stay in 419 patients. For this reason, on the one hand, it is necessary to interpret the results in light of the investigated endpoint or outcome, and on the other hand, to conduct further RCTs with subsequent pooling of the study results in meta-analyses^21^.

The fact that prehospital intubation increases the risk of tracheostomy is not surprising. Current guidelines and teaching systems for the prehospital management of severely injured patients recommend a cautious approach with a recommendation for prehospital intubation in the presence of a GCS < 9, direct injury to the airway, severe maxillofacial fractures, head or neck injury, inhalation trauma, hypercarbia, or insufficient oxygenation. Accordingly, prehospital intubation is a surrogate for impaired neurological status, relevant ventilation failing, or direct trauma to the airway^22,23^. Studies of our group as well as studies of others could show that prehospital intubation is also a risk factor for the development of pneumonia during intensive care treatment^8,24^. The risk of occurrence of pneumonia is known to increase with the duration of mechanical ventilation, which was impressively demonstrated by the detection of a cut-off point at 102 h of MV in a former study by our research group^8^. In addition, various studies show a strong positive correlation between pneumonia and the duration of mechanical ventilation, whereby the order of causality could not be conclusively clarified until today^25^. Certainly, the distinction between ventilator-associated pneumonia and pneumonia due to trauma was and is impossible, therefore this was also not attempted by us. However, the duration of MV and the occurrence of pneumonia were adjusted predictors for tracheostomy in our study. Therefore, it is reasonable that prehospital intubation represents a risk factor for tracheostomy in our study.

The performance of tracheostomy is recommended for foreseeable mechanical ventilation ≥ 7 days, although the optimal timing remains controversial. This was shown by Adriolo et al. in an 2015 Cochrane review, and since then, no convincing attempts have been made by RCTs to fill this lack of evidence^2^. Therefore, current guidelines still recommend a tracheostomy 7–10 days after admission^26^, although the development of modern tubes has extended the safe time interval to 10 days until tube-associated complications occur^27^. This was also consistent with the observed mean TTT of 10 days in our study, demonstrating the implementation of these recommendations.

Interestingly, neither thoracic injury severity nor injury severity according to ISS represented independent risk factors after the adjustment. We can only speculate about the reason for this observation. Possible causes could have been interventions, such as surgical stabilization of the thoracic wall, as well as innovative non-invasive ventilation (NIV) techniques, which negated the independent influence of thoracic trauma as a predictor of tracheostomy. In this context, Duggal et al. were able to show in a systematic review that the use of NIV for thoracic trauma patients was able to reduce complication rates and the need for intubation in various studies^28^. Moreover, a Cochrane review as well as a meta-analysis by Coughlin et al. pooled the existing evidence that thoracic wall stabilization could shorten ventilation time and reduce the risk of intubation in flail chest patients^29^ and the non-flail chest study by the Chest Wall Injury Society, a multicentre RCT, was able to demonstrate comparable results for non-flail chest patients^30^. Thoracic wall stabilizations were not included in the database. Since this intervention was rarely performed, its potential influence remains speculative.

A remarkable observation in our study was the OR of 0.49 for sepsis diagnosis. In this context, it remains open whether sepsis patients were less frequently tracheostomized or whether tracheostomized patients less frequently developed sepsis (e.g., pneumonia-associated). Due to the limitations of the registry, the causality remains open. However, studies such as those by Nseir et al. demonstrated that tracheostomy reduced the risk of ventilator-associated pneumonia. Consequently, this could be a possible explanation for the lower sepsis rate in the subgroup^31^. Another explanation could be the higher mortality in the non-tracheotomized group, which consecutively may have led to a lower sepsis prevalence. Furthermore, the variable “sepsis” could have been a collider. We did not generate a directed acyclic graph a priori, so there remains a risk of collider bias. Follow-up studies should therefore urgently clarify whether tracheostomy can reduce sepsis risk compared with endotracheal intubation and may therefore represent a prophylactic intervention in severely injured patients with relevant thoracic trauma. Conversely, if restrained use of tracheostomy can be demonstrated in sepsis patients, restrained use may represent avoidance of another *hit* to the immune system^32,33^. It is possible that the decision to perform tracheostomies was made less frequently in patients in a cytokine storm and with adequate oxygenation to avoid further elevation of inflammatory cytokines through the use of mechanical ventilation^34^. Finally, this remains an open question in our study and needs to be investigated in follow-up research.

The calculated risk score based on a reduced variable set (Table 3) from our data allows a sufficient prognosis beyond that explained by baseline factors. Overall, the T_3_P-score shows a sensitivity of 0.68 and a specificity of 0.96, which is comparable to well-established scores (e.g., qSOFA^35^). According to our study, it remains open to what extent the score is applicable to populations other than the German population; therefore, it should be validated for use in additional populations.

## Strengths and limitations

Our study has several limitations. Due to the retrospective nature of the study, drawing definitive conclusions about the clinical utility of the score is limited. Prospective evaluation of any clinical decision rule using the T_3_P-Score is warranted. Moreover, our study focused on adult patients and did not evaluate paediatric patients at risk for tracheostomy. Due to the middle-aged mean age in our study, the results have limited applicability to geriatric patients. Another limitation is the use of the inclusion criterion AIS_Thorax_. Although this is used regularly, in the future it could be replaced by alternative scoring systems (e.g. RibScore) due to improved discrimination. Sepsis and pneumonia are diseases that develop days after admission. For this reason, they are at risk of time bias. Their predictive value has to be evaluated in future studies. In contrast, we were able to investigate a large group of patients using data sets from six German level I trauma centres. We consider the resulting validity of the score to be high. Additionally, the bias that could arise from data from only one centre is excluded by the multicentre approach. Scores such as the T_3_P-Score, have been developed on the basis of national data. For this reason, the use of the score is recommended only after external validation in the respective population.

## Conclusions

The T_3_P Score predicted the need for a tracheostomy when assessed among severely injured patients with severe thoracic trauma. The predictive validity was high, and further studies should investigate the scores in different populations.

## Supplementary Information

Supplementary Information.

## Data Availability

The data are available from the corresponding author upon request.

## Abbreviations

AIS: Abbreviated injury scale
AOR: Adjusted odds ratios
BCa: Bias-corrected and accelerated
CPR: Cardiopulmonary resuscitation
ECMO: Extracorporeal membrane oxygenation
GCS: Glasgow coma scale
ICU: Intensive care unit
ISS: Injury severity score
LOS: Length of stay
MOF: Multiple organ failure
MV: Mechanical ventilation
NIV: Non-invasive ventilation
NO: Nitric oxide
OR: Odds ratio
RCT: Randomized controlled study
RISC II: Revised injury severity score II
TBI: Traumatic brain injury
TR-DGU: TraumaRegister DGU^®^
TTT: Time-to-tracheostomy
