# Supplementary material for: Predicting tracheostomy in multiple injured patients with severe thoracic injury (AIS ≥ 3) with the new T3P-Score: a multivariable regression prediction analysis
Source: Sci Rep. 2023 Feb 24;13:3260. doi: 10.1038/s41598-023-30461-x (PMC9958106; doi:10.1038/s41598-023-30461-x)
Supplement: Supplementary file 1 — Supplementary Information. [file 41598_2023_30461_MOESM1_ESM.docx]

## Implementation of the clinical database

All six hospitals participate in the nationwide TraumaRegister DGU^®^ (TR-DGU) of the German Trauma Society (Deutsche Gesellschaft für Unfallchirurgie, DGU). The TraumaRegister DGU® (TR-DGU) of the German Trauma Society was founded in 1993 with the aim of creating a multi-centre database for the pseudonymised, standardised documentation of severely injured patients for quality assurance and research.[10] Participation in TR-DGU is voluntary; however, associated hospitals are obligated to enter at least one basic dataset for quality assurance purposes.

The documentation includes detailed information on demographics, injury patterns, comorbidities, pre- and in-hospital management, course of care while in the ICU, relevant laboratory findings (including data on transfusions), and the clinical outcomes of each individual. The inclusion criteria were admission to a hospital via the emergency room with subsequent intensive care or entrance to a hospital with vital signs and death prior to admission to the ICU.

The infrastructure for documentation and data management is provided by the Academy for Trauma Surgery (AUC: Akademie der Unfallchirurgie GmbH), a company affiliated with the German Trauma Society. Scientific leadership is provided by the Committee on Emergency Medicine, Intensive Care, and Trauma Management (Sektion NIS) of the German Trauma Society. Participating hospitals submit pseudonymised data to a central database via a web-based application. For building the basic database, data was retrieved from the TR-DGU by the hospitals involved in this study, following approval from the participating hospitals and the TR-DGU. The data were complemented by a review of the patients’ records from each hospital.

## Inclusion criteria and additional data acquisition

The following inclusion criteria were applied:

- Age ≥18 years
- Admission between 2010–2014
- Primary admissions
- ICU treatment ≥1 day
- AIS_Thorax_ ≥3

Additional data were collected by each participating clinic, pseudonymised, and added to the database, among others:

- Aspiration yes/no
- Tracheostomy yes/no
- Pneumonia yes/no (CPIS > 6 or as described below), if yes: VAP/hospital acquired pneumonia (HAP)/other, and early vs. late onset
